# Supplementary material for: Dealing with Intimate Partner Violence and Family Violence in a Regional Centre of Western Australia: A Study of the Knowledge, Attitudes, and Practices of Local Social Workers
Source: Int J Environ Res Public Health. 2023 Apr 24;20(9):5628. doi: 10.3390/ijerph20095628 (PMC10178339; doi:10.3390/ijerph20095628)
Supplement: Supplementary file 1 [file ijerph-20-05628-s001.zip › ijerph-2226858-supplementary.pdf]

Social Work Survey on Responses to Family Violence

This survey aims to understand what local Social Workers understand and do in recognizing and managing clients experiencing intimate partner violence (IPV) and family violence (FV). IPV/FV is a common problem which is associated with adverse health outcomes, and can include physical, sexual, emotional, social, and financial abuse.

The results of this survey will inform the development of future IPV/FV training and education events and the referral pathways in Geraldton and the wider Midwest region. Thank you for taking the time (estimated at 10–15 minutes) to complete this survey.

---

1. Date:
2. Age:
3. Gender:
4. Area of Social Work practice:
5. Year of Qualification:
6. How many hours a week do you work on average?
7. Average number of clients you see per week:

   ☐ 0 (i.e., not seeing clients)

   ☐ < 10

   ☐ 10 –19

   ☐ 20 – 39

   ☐ over 40

8. a. During your Social Work coursework did you receive any formal education on IPV/FV?

   Yes ☐ No ☐

   b. Did you receive education or training on IPV/Family Violence during your Social Work Field Placements?

   Yes ☐ No ☐

   c. Since graduating in your profession, how much training have you had about IPV/FV? (Please tick **all** that apply)

   a. ☐ None
   b. ☐ Completed a web-based or online program
   c. ☐ Training in a workplace setting
   d. ☐ Attended a skills-based training or workshop
   e. ☐ Attended a workshop or talk by a local organization
   f. ☐ Other in-depth training >4hours
   g. ☐ Completed a post graduate university module
   h. ☐ Other (please specify) _________________________

9. Please tick the number which best describes your confidence to:

	Not Confident 1	2	Neutral 3	4	Very Confident 5
a. Ask appropriate questions if you suspect a client is experiencing IPV/FV?	☐	☐	☐	☐	☐
b. Appropriately engage with a client who discloses IPV/FV?	☐	☐	☐	☐	☐
c. Identify history, signs, and symptoms associated with IPV/FV?	☐	☐	☐	☐	☐
d. Assess the risk to a client who discloses IPV/FV?	☐	☐	☐	☐	☐
e. Help a female client who has experienced IPV/FV to create a safety plan for herself and/or her children?	☐	☐	☐	☐	☐
f. Document IPV/FV history, observations, and findings in the case notes?	☐	☐	☐	☐	☐
g. Make appropriate referrals for a woman who has experienced IPV/FV?	☐	☐	☐	☐	☐
h. Ask about and respond to the following categories of abuse in adults:					
Emotional	☐	☐	☐	☐	☐
Physical	☐	☐	☐	☐	☐
Sexual	☐	☐	☐	☐	☐
Economic	☐	☐	☐	☐	☐
Social	☐	☐	☐	☐	☐
Spiritual	☐	☐	☐	☐	☐
Reproductive control	☐	☐	☐	☐	☐
Technological	☐	☐	☐	☐	☐
Elder	☐	☐	☐	☐	☐

10. What is the strongest single risk factor for experiencing IPV/FV? (Please tick **one**)

- ☐ Age <30yrs
- ☐ Partner abuses alcohol/drugs
- ☐ Gender - Female
- ☐ Family history of abuse
- ☐ Don't know

10.1 Which one of the following is generally true about perpetrators of IPV/FV? (Tick **one**)

- ☐ They have trouble controlling their anger
- ☐ They use violence as a means of controlling their partners
- ☐ They are violent because they drink or use drugs
- ☐ Family history of abuse
- ☐ They pick fights with anyone

11. Please respond TRUE / FALSE / DON'T KNOW for the following statements:

	TRUE	FALSE	DON'T KNOW
Alcohol consumption is the greatest single predictor of the likelihood of IPV/FV.	☐	☐	☐
Women who have experienced IPV/FV are at greater risk of injury when they leave the relationship.	☐	☐	☐
Allowing a partner to be present during the consultation of a woman who has experienced IPV/FV ensures her safety.	☐	☐	☐
Gender inequality is the underlying driver of violence against women.	☐	☐	☐
Pregnant women are at higher risk for experiencing intimate partner violence.	☐	☐	☐
Strangulation injuries indicate a high risk for IPV homicide.	☐	☐	☐
Reasons for concern about IPV/FV should not be included in a woman's case notes if she does not disclose the violence	☐	☐	☐
Even if the child is not in immediate danger, practitioners have a duty of care to consider an instance of a child witnessing IPV/FV in terms of child protection.	☐	☐	☐

12. This section asks about your attitudes and opinions regarding IPV/FV.

	Strongly Disagree	Disagree	Neutral	Agree	Strongly Agree
Women who are experiencing IPV/FV could leave the relationship if they wanted to.	☐	☐	☐	☐	☐
If a woman who has experienced IPV/FV remains in the relationship after repeated episodes of violence, she must accept responsibility for the violence.	☐	☐	☐	☐	☐
Women who are experiencing IPV/FV can make appropriate choices about how to handle their situation.	☐	☐	☐	☐	☐
Being supportive of a woman's choice to remain in a violent relationship may condone the abuse.	☐	☐	☐	☐	☐
IPV/FV is a private matter and as a health professional, I should not interfere by asking about it if the client does not directly disclose.	☐	☐	☐	☐	☐
If a woman who is experiencing IPV/FV does not acknowledge the abuse, there is very little I can do to help.	☐	☐	☐	☐	☐
I am concerned about my legal obligations if a client were to disclose IPV/FV.	☐	☐	☐	☐	☐
I worry about my own safety when working with a woman who has disclosed IPV/FV	☐	☐	☐	☐	☐
Women living with IPV/FV often misuse alcohol or other drugs.	☐	☐	☐	☐	☐

13. The following questions are regarding your current practice when seeing clients who may be experiencing IPV/FV or who have disclosed abuse to you.

	Strongly Disagree	Disagree	Neutral	Agree	Strongly Agree
There is adequate private space for me to provide support for women who are experiencing or have experienced IPV/FV.	☐	☐	☐	☐	☐
I generally do not have the time to ask about IPV/FV.	☐	☐	☐	☐	☐
I tend not to ask about IPV/FV because I have little or no experience or training in this subject.	☐	☐	☐	☐	☐
I am reluctant to ask about IPV/FV because I might offend the patient or make matters worse.	☐	☐	☐	☐	☐
I have the support of colleagues in dealing with disclosure of IPV/FV.	☐	☐	☐	☐	☐
I can match interventions to a woman's readiness to change .	☐	☐	☐	☐	☐

14. Do you ask questions (either directly or indirectly) to determine the possibility of IPV/FV when seeing female clients with the following:

	Never	Sometimes	Nearly Always	Always
Physical injuries	☐	☐	☐	☐
Mental health concerns e.g., depression/anxiety, low self-esteem, self-harm, or suicidal ideation	☐	☐	☐	☐
Eating disorders	☐	☐	☐	☐
Sleeping disorders	☐	☐	☐	☐
Drug or alcohol misuse	☐	☐	☐	☐
Children with unexplained physical, behavioral, emotional, or developmental problems	☐	☐	☐	☐
Social concerns or difficulties	☐	☐	☐	☐
Homelessness	☐	☐	☐	☐
Financial Difficulties	☐	☐	☐	☐
Unemployment	☐	☐	☐	☐
History of offending	☐	☐	☐	☐
Family Breakdown	☐	☐	☐	☐

15. How many clients experiencing FV/IPV have you seen in the last 6 months? ______________________

16. In the past 6 months, which of the following actions have you taken when you identified a client experiencing IPV/FV? (Tick **all** that apply)

- ☐ Have not identified IPV/FV in the past 6 months
- ☐ Provided information (phone numbers, pamphlets, other information) to a woman
- ☐ Counseled a woman about options she may have
- ☐ Conducted a safety assessment for the woman (and children where applicable)
- ☐ Helped a woman develop a personal safety plan
- ☐ Made referrals to local services
- ☐ Other ____________________________________

17. If you have identified clients experiencing IPV/FV within the last year, please list all professionals, services, and/or organizations you have referred the client to (if any)?

Professional, Organization, or Service referred to:	
1.	4.
2.	5.
3.	6.

18. Do you feel you have adequate knowledge of referral resources in the community (including shelters or support groups) for women who have experienced IPV/FV?

☐ Yes

☐ No

19. Does your employer/workplace have a protocol for dealing with IPV/FV?

☐ Yes, and widely used

☐ Yes, and used to some extent

☐ Yes, but not used

☐ No

☐ Unsure

Please answer the following questions based on your own experiences of working in your daily practice.

20. What are the main challenges you have in discussing and managing IPV/FV with clients?

____

____

____

21. What would you recommend in order to improve the care of clients experiencing IPV/FV in Social Work practice?

____

____

____

22. Would you like further education regarding IPV/FV? ☐ YES / ☐ NO

If yes, what would be your preferred mode of delivery (e.g. workshops, online, skills based, university module etc?

____

____

If yes, what areas or topics within IPV/FV would you like to know more about?

____

____

____

____

23. Any further comments?

____

____

____

____

Thank you for completing this survey.

Please post this survey to:

Social Work Survey
WA Centre for Rural Health
PO Box 109
Geraldton WA 6531

A stamped addressed envelope is provided with this survey for your convenience
